# Comparison of *Toxoplasma gondii* Seroprevalence in Shelter Cats and Dogs during 1999–2001 and 2009–2011 in Tokyo, Japan

**DOI:** 10.1371/journal.pone.0135956

**Published:** 2015-08-18

**Authors:** Masaaki Oi, Souichi Yoshikawa, Soichi Maruyama, Sadao Nogami

**Affiliations:** 1 Department of Veterinary Medicine, Nihon University, Fujisawa, Kanagawa, Japan; 2 Department of Food Safety, Tokyo Metropolitan Institute of Public Health, Shinjuku, Tokyo, Japan; Universidade Federal do Acre (Federal University of Acre), BRAZIL

## Abstract

*Toxoplasma gondii* is an important human health concern with respect to abortion, congenital hydrocephalus, and encephalitis in immunocompromised people. Cats and dogs both are potential sources of *T*. *gondii* because they have close contact with humans. However, no epidemiological surveys have been conducted in Tokyo over the past decade. Therefore, the present study investigated and compared the seroprevalence of *T*. *gondii* infection in shelter cats and dogs during 1999–2001 and 2009–2011 in Tokyo, Japan. Serum samples were collected from 337 shelter cats and 325 shelter dogs in urban and suburban areas of Tokyo, during 1999–2001 (233 cats and 219 dogs) and 2009–2011 (104 cats and 106 dogs). *T*. *gondii* antibodies were measured in the serum samples using a commercial latex agglutination test. Data were compared using the Fisher’s exact test, and significance was indicated at *P* < 0.05. The overall seroprevalence of *T*. *gondii* infection in cats was 5.6% (13 of 233) in 1999–2001 and 6.7% (7 of 104) in 2009–2011, and that in dogs was 1.8% (4 of 219) and 1.9% (2 of 106), respectively. Significantly higher seroprevalence was observed in cats from suburban areas compared with cats in urban areas during both periods (*P* < 0.05). These results reveal that there has been little change in the feline and canine seroprevalence over the past decade, indicating that the risk of *T*. *gondii* exposure for cats and dogs in Tokyo is considerably low as the seroprevalence has reached a steady state.

## Introduction

Toxoplasmosis is a protozoan parasitic disease with a global distribution. The agent, *Toxoplasma gondii*, can infect almost all warm-blooded animals, including humans, through ingestion of oocysts excreted from cats, ingestion of cysts within intermediate hosts, or congenitally by transplacental transmission of tachyzoites [1]. Human toxoplasmosis is an important public health concern because *T*. *gondii* causes abortion in pregnant women, hydrocephalus in infants, and encephalitis in immunocompromised people with acquired immune deficiency syndrome [1]. Up to one-third of the worldwide population is estimated to be infected with the parasite [2]. In Japan, Sakikawa *et al*. [3] reported a 10.3% seroprevalence of *T*. *gondii* infection in pregnant women, and the rate of primary infection during pregnancy was 0.25%.

Cats and dogs are the most popular pet animals worldwide. Both species are potential sources of zoonotic pathogens such as *T*. *gondii* because they have close contact with humans [4]. Cats play an important role in the *T*. *gondii* lifecycle as the only animal that sheds oocysts into the environment [5]. Although dogs do not produce oocysts, they may mechanically transport oocysts from cat feces to humans because they have the olfactory capacity and habit of seeking out and rolling in foul-smelling substances contaminating their fur [6, 7].

Tokyo is a major city and has the highest population density of humans and domestic dogs in Japan. The feline population density in Tokyo is likely higher than any other city in Japan, although no accurate information is available. The risk of human exposure to *T*. *gondii* may increase in areas with a high density of cats and dogs, and the epidemiological status of the parasite in these animals in major cities requires clarification. However, no epidemiological surveys have been conducted in Tokyo over the past decade. The present study investigated and compared the seroprevalence of *T*. *gondii* infection in shelter cats and dogs during 1999–2001 and 2009–2011 in Tokyo, Japan.

## Materials and Methods

### Study area

The study area was divided into two regions, urban and suburban, according to the Tokyo district boundaries. The urban area comprised east Tokyo, including the downtown, commercial, and waterfront areas. The suburban area was located in west Tokyo and included residential and mountainous areas. According to a survey conducted by the Tokyo Metropolitan Government in 2013 [8], the percentage of green space in the urban and suburban areas was 19.8% and 67.1%, respectively.

### Ethics statement

Serum samples were collected by shelter veterinarians including one of the authors (Yoshikawa S) in accordance with the Guidelines on Research and Survey for using animals in the Tokyo Metropolitan Animal Care and Consultation Center. Approval of an ethics committee was not required for sampling because serum collection is considered a routine procedure, and the sera were collected and stored at the center before the present study was devised. The center gave written permission for the serum samples to be used in this study (Permission number: 22DOSO2350), and the study was performed in collaboration with the center and Nihon University (as per an agreement between the Tokyo Metropolitan Animal Care and Consultation Center and Nihon University for research on zoonoses, January 11, 2012).

### Serum samples

Serum samples were collected from 337 shelter cats and 325 shelter dogs at the center. The sera of 233 cats and 219 dogs were collected between April 1999 and March 2001, and the remaining samples in 104 cats and 106 dogs between April 2009 and March 2011. Blood was aseptically collected from each animal. The serum was separated by centrifugation at 1,000 *g* for 15 min and stored at -30°C until analysis. The locality, sex, age, and breed of each animal were determined using a questionnaire. Each animal was housed individually at the center.

### *Toxoplasma* serology

*T*. *gondii* antibodies were measured in each sample using a commercially available, latex agglutination test kit (Toxocheck-MT; Eiken-Kagaku, Tokyo, Japan) according to the manufacturer’s instructions with minor modifications. Each serum sample was firstly diluted 16-fold with the diluent buffer, and 25 μL of the diluted test sample was further diluted with an equal volume of buffer solution, generating serial two-fold dilutions from 1:16 to 1:1,024. *T*. *gondii* infection was diagnosed at a serum titer greater than 1:64, as described previously [9–14].

### Statistical analysis

Data were statistically analyzed with the Fisher’s exact test using JavaScript-STAR ver. 5.5.7j software (available online at http://www.kisnet.or.jp/nappa/software/star/). The seroprevalence was statistically analyzed according to locality, sex, age, and breed. Differences were considered statistically significant at *P* < 0.05.

## Results

The overall seroprevalence of *T*. *gondii* infection and the titer distribution in both species are shown in Table 1. The seroprevalence according to locality, sex, age, and breed is shown in Tables 2 and 3.

**Table 1:** Seroprevalence of *Toxoplasma gondii* infection in shelter cats and dogs in Tokyo during 1999–2001 and 2009–2011.

				No. per antibody titer				
Species	Period	No. examined	No. (%) positive	1:64	1:128	1:256	1:512	1:1,024
**Cat**	1999–2001	233	13 (5.6)	5	5	1	0	2
	2009–2011	104	7 (6.7)	0	1	3	2	1
**Dog**	1999–2001	219	4 (1.8)	0	2	0	0	2
	2009–2011	106	2 (1.9)	0	1	1	0	0

No.: number of animals

**Table 2:** Seroprevalence of *Toxoplasma gondii* infection in shelter cats in Tokyo during 1999–2001 and 2009–2011.

	1999–2001		2009–2011	
Factor	No. examined	No. (%) positive	No. examined	No. (%) positive
**Locale**				
Urban area	119	3 (2.5)^†^	60	1 (1.7)^‡^
Suburban area	114	10 (8.8)^†^	44	6 (13.6)^‡^
**Sex**				
Male	88	4 (4.5)	52	1 (1.9)
Female	145	9 (6.2)	52	6 (11.5)
**Age (years)**				
Young (≤5)	142	6 (4.2)	28	3 (10.7)
Old (>5)	91	7 (7.7)	76	4 (5.3)
**Breed**				
Purebred	23	0	6	0
Mixed breed	210	13 (6.2)	98	7 (7.1)
**Total**	233	13 (5.6)	104	7 (6.7)

^†, ‡,^ Indicates a significant difference between groups according to the Fisher’s exact test (*P* < 0.05).

No.: number of animals

**Table 3:** Seroprevalence of *Toxoplasma gondii* infection in shelter dogs in Tokyo during 1999–2001 and 2009–2011.

	1999–2001		2009–2011	
Factor	No. examined	No. (%) positive	No. examined	No. (%) positive
**Locale**				
Urban	108	1 (0.9)	60	2 (3.3)
Suburban	111	3 (2.7)	46	0
**Sex**				
Male	126	1 (0.8)	53	0
Female	93	3 (3.2)	53	2 (3.8)
**Age (years)**				
Young (≤5)	98	1 (1.0)	8	0
Old (>5)	121	3 (2.5)	98	2 (2.0)
**Breed**				
Purebred	68	1 (1.5)	72	2 (2.8)
Mixed breed	151	3 (2.0)	34	0
**Total**	219	4 (1.8)	106	2 (1.9)

No.: number of animals

In cats, the overall seroprevalence of *T*. *gondii* infection was 5.6% (13 of 233) in 1999–2001 and 6.7% (7 of 104) in 2009–2011. By locality, the seroprevalence in urban cats was 2.5% (3 of 119) in 1999–2001 and 1.7% (1 of 60) in 2009–2011, and that in suburban cats was 8.8% (10 of 114) in 1999–2001 and 13.6% (6 of 44) in 2009–2011. The seroprevalence differed significantly between urban and suburban cats during both periods (*P* < 0.05). On the other hand, no significant differences were observed in the seroprevalence between the time periods, sex, age, or breeds of cats.

In dogs, the overall seroprevalence of *T*. *gondii* infection was 1.8% (4 of 219) in 1999–2001 and 1.9% (2 of 106) in 2009–2011. There were no significant differences in the seroprevalence between the time periods, localities, sex, age, and breeds of dogs.

## Discussion

Although cats and dogs serve as potential sources of *T*. *gondii* infection for humans, the recent infection status of these animals in Tokyo, Japan is unknown. The present study demonstrated that the seroprevalence of *T*. *gondii* infection in shelter cats was 5.6% in 1999–2001 and 6.7% in 2009–2011, while that in shelter dogs was 1.8% in 1999–2001 and 1.9% in 2009–2011. There has been little change in the seroprevalence in shelter animals of either species over the past decade, indicating that the risk of *T*. *gondii* exposure for cats and dogs in Tokyo is considerably low as the seroprevalence has reached a steady state. The higher seroprevalence in cats in the present study may reflect that cats are better hunters of rodents and small birds than dogs [15] and thus have higher opportunity to be infected with *T*. *gondii*. The seroprevalence of *T*. *gondii* infection is known to increase with age in cats and dogs [16, 17]. Notably, the seroprevalence in young cats in 2009–2011 was more than twice the seroprevalence in older cats, though this difference was not significant. Moreover, the young cats had higher titers, ranging from 1:256 to 1:1,024, which indicates recent infection, frequent infection, or re-infection by the parasite. On the other hand, the lower titers in older cats suggest latent infection. *T*. *gondii* infection appears to be more prevalent in the young feline population in Tokyo, though the reason for this is unclear.

The feline seroprevalence (5.6–6.7%) in the present study is similar to those seen in previous surveys conducted in Japan after 1998, in which the seroprevalence ranged from 5.4% to 8.7% among domestic cats [8, 10, 11]. The latest study (2005) also reported a seroprevalence of 5.7% in 1,327 domestic cats from the Kanto region [12]. Similarly, little differences were observed in the canine seroprevalence (1.8–1.9%) in the present study compared to that in a previous survey (2008) with a seroprevalence of 1.7% among 519 domestic dogs from the same region [13]. These data indicate exposure of both cats and dogs to *T*. *gondii* and widespread contamination of the parasite in human environments.

The seroprevalence of *T*. *gondii* infection in cats varies by geographic location [14]. It can differ within specific areas of a country and within a single city [1]. Suburban and rural cats are more likely to be infected with *T*. *gondii* than urban cats [18]. In the present study, the seroprevalence was significantly higher in suburban cats during both periods than that in urban cats. This indicates that suburban cats had more contact with infection sources, such as infected cats, intermediate hosts, and soil or water contaminated with *T*. *gondii* oocysts [1]. The difference in the seroprevalence between the localities may depend upon the density of outdoor cats, density of intermediate hosts, and dietary habits of cats. Outdoor access and hunting behavior are recognized as risk factors for *T*. *gondii* infection in cats [16, 19, 20]. Alternately, there is much more green space in suburban areas than in urban areas (67.1 vs. 19.8%), and, consequently, oocysts may survive for longer periods in the former because urban oocysts may be more easily washed away from cement surfaces by rain or face greater exposure to direct sunlight [21]. The seroprevalence is also reportedly higher in rural dogs than in urban dogs [22, 23]. However, in the shelter dogs of the present study, the seroprevalence of *T*. *gondii* infection was not significantly associated with the locale. Because most dogs in the present study were former pets with less chance of roaming freely outside, no locality-related differences were apparent.

Our results revealed a low seroprevalence of *T*. *gondii* infection in both shelter cats and dogs in Tokyo over the past decade. Seropositive cats are thought to excrete large numbers of *T*. *gondii* oocysts that contaminate the human environment. Given the significantly higher seroprevalence observed in suburban cats, residents in these areas may be at high risk of exposure to *T*. *gondii* through environmental contamination with cat feces. Although the role of dogs in *T*. *gondii* infection has generally been considered of secondary importance [22], cohort studies reported that the risk of *T*. *gondii* exposure in children as a result of contact with dogs was greater than in children in contact with cats [24, 25]. In particular, children, pregnant women, and immunocompromised people should adhere to hygienic principles not only after contact with soil and cats, but also after contact with dogs.
